# Peripherin is a biomarker of axonal damage in peripheral nervous system disease

**DOI:** 10.1093/brain/awad234

**Published:** 2023-07-12

**Authors:** Stephen Keddie, Duncan Smyth, Ryan Y S Keh, Michael K L Chou, Donna Grant, Sunaina Surana, Amanda Heslegrave, Henrik Zetterberg, Luuk Wieske, Milou Michael, Filip Eftimov, Roberto Bellanti, Simon Rinaldi, Melanie S Hart, Axel Petzold, Michael P Lunn

**Affiliations:** Department of Neuromuscular Diseases, Barts Health NHS Trust, London E1 1BB, UK; Department of Neuromuscular Diseases, University College London, London WC1N 3BG, UK; Centre for Neuromuscular Disease, National Hospital for Neurology and Neurosurgery, London WC1N 3BG, UK; Department of Neuromuscular Diseases, University College London, London WC1N 3BG, UK; Centre for Neuromuscular Disease, National Hospital for Neurology and Neurosurgery, London WC1N 3BG, UK; Department of Neuromuscular Diseases, University College London, London WC1N 3BG, UK; Centre for Neuromuscular Disease, National Hospital for Neurology and Neurosurgery, London WC1N 3BG, UK; Department of Neuromuscular Diseases, University College London, London WC1N 3BG, UK; NHS Neuroimmunology and CSF Laboratory, Queen Square Institute of Neurology, London WC1N 3BG, UK; NHS Neuroimmunology and CSF Laboratory, Queen Square Institute of Neurology, London WC1N 3BG, UK; Department of Neuroinflammation, University College London, London WC1N 3BG, UK; Department of Neuromuscular Diseases, University College London, London WC1N 3BG, UK; UK Dementia Research Institute, University College London, London WC1E 6BT, UK; Department of Neurodegenerative Disease, UCL Institute of Neurology, London WC1N 3BG, UK; UK Dementia Research Institute, University College London, London WC1E 6BT, UK; Department of Neurodegenerative Disease, UCL Institute of Neurology, London WC1N 3BG, UK; Hong Kong Center for Neurodegenerative Diseases, Hong Kong, China; Wisconsin Alzheimer’s Disease Research Center, University of Wisconsin School of Medicine and Public Health, University of Wisconsin-Madison, Madison, WI 53792, USA; Department of Psychiatry and Neurochemistry, Institute of Neuroscience and Physiology, The Sahlgrenska Academy at the University of Gothenburg, Mölndal 431 41, Sweden; Clinical Neurochemistry Laboratory, Sahlgrenska University Hospital, Mölndal 431 41, Sweden; Department of Neurology and Neurophysiology, Amsterdam Neuroscience, Amsterdam UMC, Location AMC, University of Amsterdam, 1081 HV Amsterdam, The Netherlands; Department of Neurology and Neurophysiology, Amsterdam Neuroscience, Amsterdam UMC, Location AMC, University of Amsterdam, 1081 HV Amsterdam, The Netherlands; Department of Neurology and Neurophysiology, Amsterdam Neuroscience, Amsterdam UMC, Location AMC, University of Amsterdam, 1081 HV Amsterdam, The Netherlands; Nuffield Department of Clinical Neurosciences, University of Oxford, Oxford OX3 9DU, UK; Nuffield Department of Clinical Neurosciences, University of Oxford, Oxford OX3 9DU, UK; NHS Neuroimmunology and CSF Laboratory, Queen Square Institute of Neurology, London WC1N 3BG, UK; Department of Neuroinflammation, University College London, London WC1N 3BG, UK; Department of Neurology and Neurophysiology, Amsterdam Neuroscience, Amsterdam UMC, Location AMC, University of Amsterdam, 1081 HV Amsterdam, The Netherlands; UCL Clinical and Movement Neurosciences Department, National Hospital for Neurology and Neurosurgery, UCL Institute of Neurology, London WC1E 6BT, UK; Department of Neuromuscular Diseases, University College London, London WC1N 3BG, UK; Centre for Neuromuscular Disease, National Hospital for Neurology and Neurosurgery, London WC1N 3BG, UK; NHS Neuroimmunology and CSF Laboratory, Queen Square Institute of Neurology, London WC1N 3BG, UK

**Keywords:** peripherin, axon, biomarker, guillain-Barré syndrome, neuropathy, peripheral nervous system

## Abstract

Valid, responsive blood biomarkers specific to peripheral nerve damage would improve management of peripheral nervous system (PNS) diseases. Neurofilament light chain (NfL) is sensitive for detecting axonal pathology but is not specific to PNS damage, as it is expressed throughout the PNS and CNS. Peripherin, another intermediate filament protein, is almost exclusively expressed in peripheral nerve axons. We postulated that peripherin would be a promising blood biomarker of PNS axonal damage.

We demonstrated that peripherin is distributed in sciatic nerve, and to a lesser extent spinal cord tissue lysates, but not in brain or extra-neural tissues. In the spinal cord, anti-peripherin antibody bound only to the primary cells of the periphery (anterior horn cells, motor axons and primary afferent sensory axons). *In vitro* models of antibody-mediated axonal and demyelinating nerve injury showed marked elevation of peripherin levels only in axonal damage and only a minimal rise in demyelination.

We developed an immunoassay using single molecule array technology for the detection of serum peripherin as a biomarker for PNS axonal damage. We examined longitudinal serum peripherin and NfL concentrations in individuals with Guillain-Barré syndrome (GBS, *n* = 45, 179 time points), chronic inflammatory demyelinating polyradiculoneuropathy (CIDP, *n* = 35, 70 time points), multiple sclerosis (*n* = 30), dementia (as non-inflammatory CNS controls, *n* = 30) and healthy individuals (*n* = 24).

Peak peripherin levels were higher in GBS than all other groups (median 18.75 pg/ml versus < 6.98 pg/ml, *P* < 0.0001). Peak NfL was highest in GBS (median 220.8 pg/ml) and lowest in healthy controls (median 5.6 pg/ml), but NfL did not distinguish between CIDP (17.3 pg/ml), multiple sclerosis (21.5 pg/ml) and dementia (29.9 pg/ml). While peak NfL levels were higher with older age (*rho* = +0.39, *P* < 0.0001), peak peripherin levels did not vary with age.

In GBS, local regression analysis of serial peripherin in the majority of individuals with three or more time points of data (16/25) displayed a rise-and-fall pattern with the highest value within the first week of initial assessment. Similar analysis of serial NfL concentrations showed a later peak at 16 days. Group analysis of serum peripherin and NfL levels in GBS and CIDP patients were not significantly associated with clinical data, but in some individuals with GBS, peripherin levels appeared to better reflect clinical outcome measure improvement.

Serum peripherin is a promising new, dynamic and specific biomarker of acute PNS axonal damage.

## Teaser

Keddie *et al*. have developed a highly sensitive immunoassay to measure the intermediate filament protein peripherin in the blood. Peripherin can be used as a biomarker of specific damage to the peripheral nervous system, helping with identification, prognostication and treatment of peripheral nerve diseases.

## Introduction

The measurement of disease activity and treatment response in peripheral neuropathies is broadly limited to physical examination, clinical assessment scores and neurophysiological testing. These techniques require significant time investment from skilled operators, often lack inter-observer reliability and are limited by uncontrolled external factors. Valid and responsive fluid biomarkers are needed to improve the accuracy of diagnosis, prognostication and monitoring of disease. In neurology, neurofilament light (NfL) has become the exemplar.

NfL is a type IV intermediate filament of the axonal cytoskeleton and is expressed ubiquitously in the neurons of the peripheral (PNS) and CNS. NfL rises with reasonable sensitivity in almost all neurological diseases; at least 87 diseases in neurology have data indicating potentially ‘useful’ rises in NfL with neuronal damage.^1,2^ Quantification of NfL therefore lacks specificity, where raised NfL levels cannot indicate the type of neurological disorder or differentiate between peripheral versus centrally derived axonal damage. In addition, it is unclear how well it follows the time course of disease and recovery in peripheral nerve injuries such as Guillain-Barré syndrome (GBS) and chronic inflammatory demyelinating polyradiculoneuropathy (CIDP).^3^

The axonal cytoskeleton has three types of filament proteins, those being microtubules, microfilaments and intermediate filaments, all of which are released in axonal injury. Intermediate filaments are classified into five subtypes (I–V) of which peripherin is a type III intermediate filament protein expressed almost exclusively in peripheral nerve dorsal root ganglia, ventral motor neurons, and the cranial nerves.^4,5^ In sciatic nerves, the distribution and abundance of peripherin in peripheral nerves is essentially identical to the other triplet neurofilament proteins (neurofilament light, medium and heavy).^6^ The physiological function of peripherin is unknown, but high expression during development and outgrowth of axons following axotomy injuries suggest roles in axonal regeneration and guidance.^7,8^ The almost complete specificity of peripherin to peripheral nerves coupled with similar abundance to NfL makes peripherin a promising biomarker candidate for peripheral nerve axonal damage.

A single study of peripherin levels using a commercial enzyme-linked immunoassay (ELISA) in patients with motor neuron disease (MND) reported serum peripherin levels were increased in MND compared to control subjects.^9^ However, the levels of peripherin reported were much higher than expected (measured in ng/ml rather than pg/ml). The study found no difference between patients with peripheral neuropathy and healthy controls, which may be due to a lack of sensitivity using ELISA and the requirement of more ultrasensitive techniques.

We have developed and optimized an ultrasensitive immunoassay for the quantification of serum peripherin as a biomarker for peripheral nerve axonal damage. We evaluated (i) the distribution of peripherin throughout neuronal and non-neuronal organs; and whether (ii) peripherin was released into myelinated co-cultures with axonal damage; (iii) could differentiate PNS from CNS disease and healthy controls; (iv) longitudinally correlate with disease; and (v) differentiate peripheral nerve disorders based upon the degree of axonal loss.

## Materials and methods

### SDS-PAGE and immunoblot analysis

We explored the distribution of peripherin and neurofilament in a range of neuronal and non-neuronal tissues. 

Multiple organs from p56 Sprague Dawley rats were harvested. Tissues were mechanically homogenized on ice in 4× weight/volume of lysis buffer containing 60 mM Tris-HCl, pH 6.8, 2% sodium dodecyl sulphate (SDS) and 10% sucrose with 1:100 protease inhibitor (P8340 Sigma-Aldrich). Samples were centrifuged at 21 000*g* (15 000 rpm) for 30 min at 4°C. Supernatant homogenate protein concentrations were determined with bicinchoninic acid (BCA) assay (23225 Thermo Fisher Scientific). Tissue homogenate (10 μg) was loaded on 4–15% Mini-PROTEAN TGX Stain-Free Protein Gels (Bio-Rad). Samples were migrated at 120 V for 10 min then 50 min at 150 V, followed by a semi-dry transfer to PVDF membrane for 30 min. Primary monoclonal antibodies (mAbs) NfL (N5139, Sigma-Aldrich), peripherin (P5117, Sigma-Aldrich), GAPDH (MAB374, Merck) were diluted to 1:1000 in PBS 1% bovine serum albumin (BSA) and added to an overnight incubation at 4°C. Secondary rabbit anti-mouse immunoglobulin/HRP (P0260 Agilent) at 1:1000 in PBS 1% BSA was incubated for 2 h at room temperature before electrochemiluminescent development using a ChemiDoc MP Imaging System (Bio-Rad).

### Frozen spinal cord sectioning for immunohistochemistry

The spinal column from p56 Sprague Dawley rats was extracted and dissected into 1 cm sections, spinal cord was then removed from the spinal column and snap frozen with liquid nitrogen. Snap-frozen material was stored at −80°C until needed to be sectioned. For use, thoracic spinal cord was dissected to 0.2 mm transverse thicknesses, sectioned to 10 µm and attached to Polysine slides (631-0107, Avantor).

Immunostaining was performed at room temperature. Sections were blocked in 5% BSA for 30 min. Primary anti-peripherin antibody (sc-377093, Santa-Cruz) and neurofilament light chain antibody (MA5-14981, Thermo Fisher) were applied for 30 min at 1:1000 dilution with 1% BSA and then washed three times with PBS, each for 10 min. Secondary antibodies [goat anti-mouse Alexa 488 (A-11001, Thermo Fisher) and donkey anti-rabbit Alexa 555 (A-31572)] at 1:1000 dilution in 1% BSA-PBS were added, incubated for 30 min, and then washed with PBS. Sections were imaged using a NanoZoomer S60 Digital scanner (model C13210-01). Haematoxylin and eosin (H&E) staining was performed for structure and section quality assessment (not shown).

### Development of Simoa immunoassay

Candidate single molecule array (Simoa) anti-peripherin primary and secondary mAbs were tested in a checkerboard titration with full-length human recombinant peripherin protein (NM_006262, Origene) to determine the antibody combination with the best signal-to-noise ratio across a number of peripherin concentrations ranging from 1 to 10 000 pg/ml (results not shown).

The selected antibodies were bead coupled or biotinylated for use in Simoa as per the Simoa Homebrew Assay Development Guide (Quanterix).^10^ In short, capture antibodies were prepared by buffer exchange into the Quanterix recommended bead conjugation buffer using Amicon Ultra-0.5 50 kDa centrifugal filters (Merck) following manufacturer recommendations. Paramagnetic carboxylated beads (103 207; Quanterix) were washed and prepared to provide a supply of 1.4 × 10^9^ beads/ml of capture antibody solution. Conjugation of the capture antibodies to beads using 1-ethyl-3-(3-dimethylaminopropyl)carbodiimide (EDC) chemistry was performed according to Quanterix® manual protocol, and tested using a range of antibody and EDC concentrations (see later). Beads were placed on a rollator at 2–8°C for 120 min (HulaMixer, Thermo Fisher) to conjugate. Conjugated beads were washed, blocking solution was added and then incubated at 2–8°C for 45 min. Following three washes, the conjugated beads were resuspended and stored at 4°C pending use.

The detection antibodies were buffer-exchanged into Quanterix® biotinylation reaction buffer using Amicon Ultra-0.5 50 kDa filters. Antibody concentration was adjusted to between 0.3–2 mg/ml with biotinylation reaction buffer prior to conjugation to 8.9 mM NHS-PEG4-biotin (A3959: Thermo Fisher Scientific) at various ratios (×20, × 40 or ×60) and incubated for 30 min at room temperature. Unlabelled surplus material was removed through additional Amicon filter buffer exchange.

Each capture antibody was made to test an initial 0.3 ml lot of beads with 0.2 mg/ml antibody and 0.3 mg/ml EDC with conjugation performed at 2–8°C. Antibody combinations (capture and detector) were then tested to detect recombinant peripherin protein (NM_006262, Origene) from a standard curve. The capture and detector combination with optimum signal:noise [8G2 capture mAb (Sigma-Aldrich) and detector mAb A-3 anti-peripherin mouse monoclonal (Santa-Cruz)] was further optimized.

The final optimized two-step assay was performed on a Simoa HD-X (Quanterix). 8G2 was coated to carboxylated paramagnetic beads at a concentration of 0.3 mg/ml and 350 000 assay beads and 150 000 helper beads were added per cuvette at 2 × 10^7^ beads/ml. The detector mAb was biotinylated at ×40 with 8.9 mM NHS-PEG4-biotin (A3959: Thermo Fisher Scientific). Detector antibody (20 μl) and 100 μl of sample (or calibrator) were added to the bead pellet. Samples were pre-diluted 1:8 with sample diluent A (Quanterix) + Triton X 0.5%. Following a 47-cadence incubation (one cadence = 45 s), the beads were washed, followed by addition of 100 μl of 50 pM streptavidin-conjugated β-galactosidase (Quanterix). This was followed by a seven-cadence incubation and a wash. Before reading, 25 μl resorufin β−D-galactopyranoside (RGP, Quanterix) was added.

A calibration curve was optimized from 0 to 9000 pg/ml, expected to cover the likely range of serum peripherin concentrations while maintaining sensitivity. Further optimization steps were performed as part of the recommended assay development guideline. The optimal assay conditions were iteratively tested with the best assay parameter being selected and then kept constant for subsequent assays. A representative assay calibration curve is depicted in Supplementary Fig. 1. The lower limit of detection (LLOD) was defined as 2.5 times the standard deviation (SD) plus the mean average enzymes per bead (AEB) signal of six replicate results of the blank calibrator. This was 0.872 pg/ml, corrected to an absolute value of 6.98 pg/ml when taking into account the 1:8 dilution. Assay validation experiments are included in the Supplementary material.

### Myelinating cell co-culture antibody-mediated injury, staining and confocal imaging

Myelinating cell co-cultures were prepared using human induced pluripotent stem cell (hiPSC)-derived sensory neurons and primary rat Schwann cells as previously described.^11^ HiPSCs from control subjects were obtained through the IMI/EU sponsored StemBANCC consortium via the Human Biomaterials Resource Centre, University of Birmingham, UK (http://www.birmingham.ac.uk/facilities/hbrc). Axonal injury was induced using 14G2A anti-GD2 monoclonal antibody at 10 μg/ml in neurobasal media supplemented with 1× N2 (Cat. No. 17502-048), B27 (Cat. No. 12587-010), Glutamax (Cat. No. 35050-038) and 1× antibiotic-antimycotic (penicillin, streptomycin and amphotericin) mixture (Cat. No. 15240-062) (all Gibco, Life Technologies) (‘complete’ neurobasal medium) plus recombinant human β-NGF (rhNGF) at 25 ng/ml (Cat. No. 450-01, Peprotech). To induce demyelination, serum with previously established IgG myelin reactivity was used in place of the 14G2A anti GD2 monoclonal antibody.^12^ One hour after incubation with antibody or serum, normal human serum 20% was added to induce complement-mediated demyelination and axonal injury. Culture supernatants were collected from two wells per condition (control, axonal damage, demyelination) at each time point (before injury, 4 h, 24 h). For immunocytochemistry, coverslips were transferred to PBS, fixed in 4% paraformaldehyde for 30 min, washed three times in PBS then permeabilized in ice cold methanol for 20 min. Following three PBS washes, cells were blocked using PBS with 5% normal goat serum (NGS), washed in PBS and incubated with primary antibodies (chicken anti-NF200 antibodies at 1:10 000 for axons and rat anti-myelin basic protein MBP antibodies at 1:500 for myelin) overnight at 4°C. Cells were then washed with PBS and incubated with the secondary antibodies [goat anti-chicken Biotin (1:500) Life Tech BA9010, and goat anti-rat Alexa Fluor 546 (1:1000) Life Tech A11081] for 1 h on orbital shaker (500 rpm) covered in tin foil. The secondary antibody was washed off with PBS, followed by incubation with Streptavidin-Pacific Blue (1:500, Life Tech S11222). Finally, the coverslips were mounted onto Superfrost Plus microscope slides (Thermo Scientific) in Vectashield mounting medium (Vector Laboratories). Confocal microscopy was used to confirm axonal degeneration and quantify myelination, and objective sampling across the coverslip was ensured through systematic random sampling. Images were acquired on a Leica TSC SP5 confocal microscope.

### Patient clinical assessment and sample testing

Serial longitudinal blood samples were collected from 45 patients with GBS (179 time points) and 35 treatment-naïve patients with CIDP prior to induction therapy (70 time points). All patients had been evaluated by specialist neuromuscular neurologists confirming the clinical diagnosis (Table 1). Clinical diagnostic certainty was classified according to the Brighton diagnostic criteria for GBS, and for CIDP according to the 2010 European Federation of Neurological Societies/Peripheral Nerve Society (EFNS/PNS) diagnostic criteria for CIDP.^13,14^ The majority of GBS patients (34/45, 76%) had multiple clinical samples taken at different time points. The exact time points varied between patients, but the majority of GBS patients with multiple samples had these taken at baseline and at Weeks 1, 2 and 4, with some having additional samples at later time points up to and beyond 1 year from onset. CIDP patients had two samples taken between 6 weeks and 12 months apart (18 months in one case).

**Table 1:** Baseline cohort demographics

	GBS	CIDP	MS	Dementia	HC
Total case number (*GBS cases with associated clinical information*)	45 (*25*)^a^	35	30	30	24
Time points (*GBS time points with associated clinical information*)	179 (*52*)^a^	70	30	30	24
Median age (SD)	45 (15.4)	64 (12.7)	36 (14.1)	66 (5.6)	35 (10.5)
Gender (% female)	48%	31%	63%	47%	50%
Median follow-up duration (days, SD)	33 (157.5)	176 (95.8)	N/A	N/A	N/A
**GBS cohort characteristics (*n* = patients with available data)**			***n* (% or SD)**		
Median time (in days) from symptom onset to first assessment (*n* = 25)			17 (10.7)		
Median time (in days) to nadir (*n* = 21)			11 (10.1)		
Prior infection reported (*n* = 21)			11 (52%)		
Respiratory compromise requiring intubation (*n* = 25)			5 (20%)		
Hughes GBS score on first assessment (*n* = 21)					
0, Normal			0		
1, Slight clinical symptoms/signs			1 (5%)		
2, Able to walk 5 m unaided, unable to run			2 (10%)		
3, Able to walk 5 m with help			5 (24%)		
4, Bedridden/chairbound			11 (52%)		
5, Ventilator-assisted breathing			2 (10%)		
Brighton Criteria classification (*n* = 21)					
Level 1 (highest certainty)			7 (33%)		
Level 2			11 (52%)		
Level 3			2 (10%)		
Level 4 (lowest certainty)			1 (5%)		
Nerve conduction studies (*n* = 21)					
Demyelinating			14 (67%)		
Axonal			4 (19%)		
Mixed			2 (10%)		
Equivocal			1 (5%)		
Median mEGOS score at Day 7 (*n* = 21)			5 (3.9)		
Median iRODS at first assessment (*n* = 21)			6 (13.6)		
Median ONLS at nadir (*n* = 21)			8 (3.6)		
Median MRC-SS at nadir (*n* = 21)			37 (22)		
Treatment (*n* = 21)					
Intravenous immunoglobulin			18 (86%)		
Plasma exchange			2 (10%)		
**CIDP cohort characteristics (*n* = patients with available data)**			***n* (% or SD)**		
CIDP subtype (*n* = 35)					
Typical			19 (54%)		
Multifocal			8 (23%)		
Distal			4 (11%)		
Sensory/sensory predominant			3 (9%)		
Motor/motor predominant			1 (3%)		
Treatment (*n* = 35)					
Corticosteroids only			7 (20%)		
Intravenous immunoglobulin only			3 (9%)		
Intravenous immunoglobulin + corticosteroids			13 (37%)		
OPTIC trial (IVIG + steroids or placebo)			12 (34%)		
Median iRODS at baseline (*n* = 35)			34 (12.1)		
Median MRC-SS at baseline (*n* = 35)			54 (6.0)		
Median iRODS on follow-up (*n* = 35)			39 (11.3)		
Median MRC-SS on follow-up (*n* = 35)			58 (6.7)		

CIDP = chronic inflammatory demyelinating polyradiculoneuropathy; F = female; GBS = Guillain-Barré syndrome; HC = healthy controls; iRODS = Inflammatory Rasch Built Outcome Score; mEGOS = modified Erasmus Guillain-Barré Outcome Score; MRC-SS = Medical Research Council Sum Score; MS = multiple sclerosis; N/A = non applicable; ONLS = Overall Neuropathy Limitation Scale; SD = standard deviation.

Clinical data were available for 25 of 45 cases.

Single blood samples were also obtained from 24 healthy individuals (negative controls), 30 individuals with multiple sclerosis (inflammatory CNS controls) and 30 people with dementia (non-inflammatory CNS controls).

Blood samples were centrifuged at 3000*g* for 5 min and snap frozen at −80°C. Serum samples were analysed for peripherin as described above. Two internal controls (serum samples with high and low peripherin levels) were used for internal quality control between runs. Sera from 55 GBS samples at various time points were analysed using a smaller amount of detection antibody than in the assay protocol (1.3 instead of 2.1 μg/ml). This resulted in lower concentrations of the internal control samples, so an internal corrective scaling was used for these patient samples from the internal quality controls. Serum samples were also tested for NfL using the Quanterix NF-light® Advantage Kit.^15^

Clinical data were available for 25 of the 45 GBS patients, including demographic information, symptoms at onset, clinical examination, Medical Research Council Sum Score (MRC-SS), inflammatory-RODS (i-RODS),^16^ Overall Neuropathy Limitations Scale (ONLS),^17^ modified Rankin scale (mRS), investigations and treatment details. For CIDP patients, disease phenotype, MRC-SS, i-RODS and treatment details were obtained.

### Statistical analysis

Statistical analysis was performed using Graphpad Prism v8.1 (San Diego, CA, USA) and R v4.0.3 (R Core Team) was used for analysis.

Mann-Whitney U and Kruskal-Wallis tests were used to compare demographic and clinical data between the different groups.

For GBS and CIDP, peak biomarker levels were used for the correlation analysis because of the likelihood of different patterns of rise and fall between peripherin and NfL, and the fact that peak levels likely reflect the greatest extent of axonal damage.

Kruskal-Wallis and Dunn’s multiple comparisons tests were used to compare peak peripherin and peak NfL levels between the different disease groups. Spearman correlation coefficient was used to determine correlation between peak serum peripherin and peak NfL levels in each of the different groups, and to compare peak peripherin and NfL levels with age.

The LLOD of the peripherin assay was 0.87 pg/ml, which meant that the lower limit of detection for samples was 6.98 pg/ml (samples diluted 1:8). When comparing peripherin levels between different groups, the samples with peripherin below the LLOD were assumed to have a level of 6.98 pg/ml, to reduce the risk of finding a spurious association (type I error).

The Spearman’s correlation coefficient, Kruskal-Wallis test and Mann-Whitney U-test were used as appropriate to compare peak and longitudinal peripherin and NfL levels with GBS and CIDP patient demographics, disease phenotype, investigations and treatment details.

Peripherin levels of the 25 GBS patients with more than three samples were graphed longitudinally, to assess the temporal pattern of peripherin and NfL with time. A receiver operating characteristic (ROC) curve was used to assess the diagnostic utility of peak serum peripherin and NfL to identify GBS as opposed to CIDP, using Graphpad and the pROC package in R. A binomial GLM regression model was also used to investigate the diagnostic ability of peak serum peripherin combined with peak serum NfL for differentiating GBS and CIDP.

A significance value of *P* < 0.05 was used throughout, with Bonferroni adjustment for multiple comparisons where appropriate.

### Ethics

Collection and analysis of GBS and CIDP patient samples was approved by London- Queen Square Research Ethics Committee (16/LO/1852), and Amsterdam UMC (METC AMC 2015_254, METC AMC 2014_132). Written informed consent was obtained from all participants in the study. Pseudonymized samples from individuals in the multiple sclerosis, dementia and healthy control groups were analysed exempt of the need for consent under the Human Tissue Act 2004 for assay quality assurance.^18^

## Results

### Distribution of peripherin in neuronal and non-neuronal tissues

**Figure 1:**
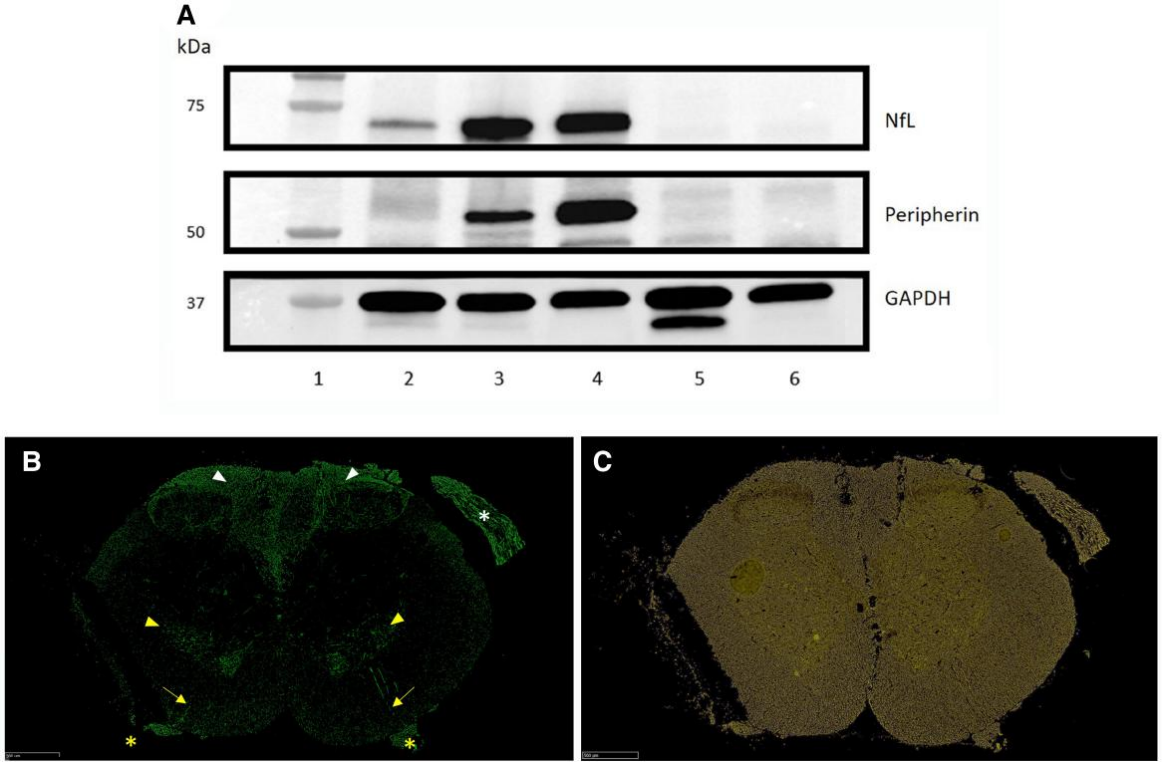
**Western blot and immunohistochemical staining demonstrating tissue distribution of peripherin within the nervous system**. (**A**) Western blot of neuronal and non-neuronal tissue lysates demonstrating differential distribution of peripherin and neurofilament light (NfL) within the nervous system. Lane 1: molecular weight marker. NfL is detected in brain (Lane 2), spinal cord (Lane 3) and sciatic nerve (Lane 4) tissue lysates, whereas peripherin is not detected in the brain, and less strongly within the spinal cord than the sciatic nerve. Neither NfL nor peripherin are detected within heart (Lane 5) or liver (Lane 6) tissue. (**B** and **C**) Frozen mouse thoracic spinal cord sections. Immunohistochemical staining with peripherin antibody (**B**) demonstrates anti-peripherin antibody binding to anterior horn cells (yellow arrowheads), exiting alpha motor neurons (yellow arrows), central axon of primary pseudobipolar sensory nerves in dorsal column (white arrow heads) and dorsal (white asterisk) and ventral roots (yellow asterisks). Neurofilament (**C**) stains all axons in cord and dorsal and ventral roots.

NfL is present in the brain and spinal cord (CNS) and in the PNS. Peripherin is not measurably present in the brain, is detectable in the spinal cord and most abundant in peripheral nerve axons. Figure 1A demonstrates the distributions of NfL and peripherin in nervous system and in non-neurological tissues. Peripherin detected in spinal cord lysate is derived from anterior horn cells, exiting alpha motor neurons, the primary afferent sensory neurons in the dorsal columns, and in the dorsal and ventral roots rather than from spinal axons, whereas NfL was detected in all spinal cord axons including dorsal and ventral roots. This is demonstrated immunohistochemically in spinal cord tissue in Fig. 1B and C.

### Cell culture

**Figure 2:**
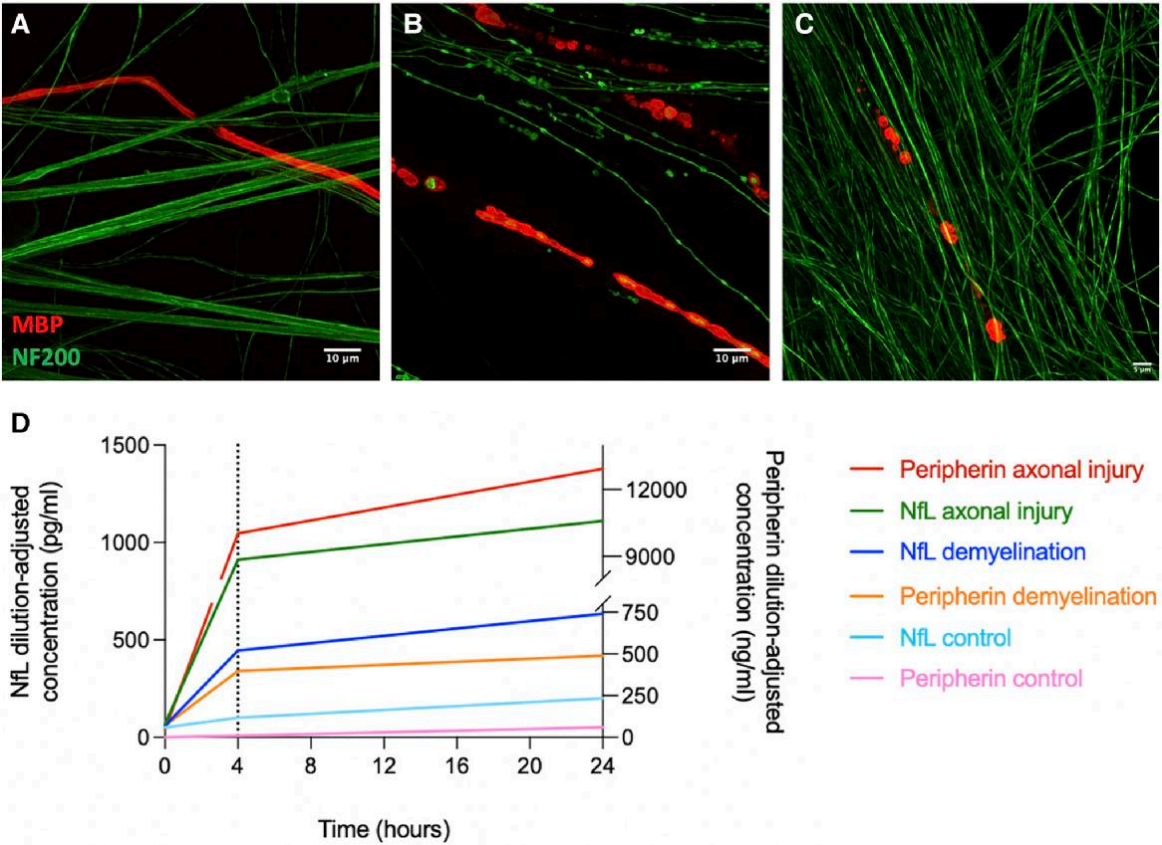
***In vitro* models of antibody-mediated axonal injury and demyelination.** Culture systems with human iPSC-derived neurons and rodent Schwann cells. (**A**) Control culture without damage. (**B**) A reduction in axonal density, irregular axonal staining and blebbing (NF200, green) are seen after immunological injury to the axons. Secondary myelin breakdown is also seen (MBP, red). (**C**) Primary demyelination with preserved axonal structure and myelin blebbing. (**D**) Simoa measurements of NfL and peripherin in culture supernatants at baseline, 4 and 24 h. Axonal degeneration associated with substantially higher supernatant levels of NfL and peripherin compared to demyelination and control supernatants. Levels increase rapidly and rise over 24 h. iPSC = induced pluripotent stem cell; NfL = neurofilament light chain.

Antibody-mediated axonal injury and demyelination were initially confirmed and assessed with immunocytochemistry and confocal microscopy (Fig. 2A–C). Simoa was then used to measure peripherin levels in the supernatants from two co-cultures per condition (primary axonal damage, primary demyelination and controls), diluted 1/100 with sample diluent to bring into the assay’s measurable range. Twenty-four hours following injury, peripherin levels were significantly higher in the supernatants with axonal damage versus demyelination (12.93 × 10^6^ versus 0.48 × 10^6^ pg/ml, *P* = 0.0031) and control conditions (12.93 × 10^6^ versus 0.14 × 10^6^ pg/ml, *P* = 0.0029), demonstrated in Fig. 2D. Differences in peripherin levels at 24 h from injury were larger than differences in NfL levels (NfL axonal versus demyelination 1.1 × 10^3^ pg/ml versus 0.6 × 10^3^ pg/ml, *P* = 0.0108; axonal versus control 1.1 × 10^3^ versus 0.2 × 10^3^ pg/ml, *P* = 0.0032).

### Serum peripherin and neurofilament light measurement using Simoa immunoassay in clinical samples

Table 1 summarizes the baseline demographics of the clinical cohort.

**Figure 3:**
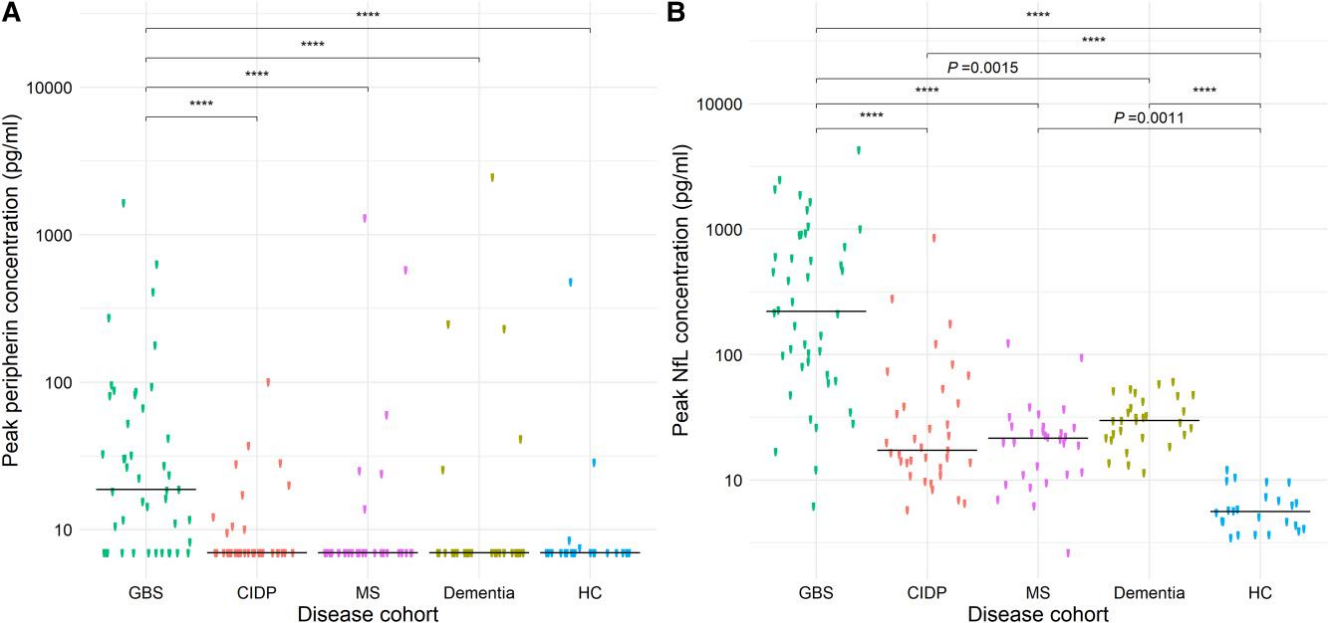
**Distribution of peak serum peripherin and neurofilament light (NfL) concentrations in different disease groups.** For Guillain-Barré syndrome (GBS) and chronic inflammatory demyelinating polyradiculoneuropathy (CIDP) patients, the highest measured serum peripherin or neurofilament light chain (NfL) concentration was taken as the peak value. Multiple sclerosis (MS) and dementia patients and healthy control subjects (HC) provided single time point samples. (**A**) Peak serum peripherin and (**B**) peak serum NfL is elevated in GBS compared to all other disease groups. Peak NfL is highest in GBS and lowest in controls, but does not distinguish between CIDP, multiple sclerosis and dementia patients. ****P < 0.0001.

Figure 3 demonstrates the differing distribution of peak serum peripherin and NfL levels within the different patient groups. Peak peripherin levels were higher in GBS (median 18.75 pg/ml) compared to each other disease group, where median peripherin was less than the LLOD of 6.98 pg/ml (*P* < 0.0001). Peak NfL was also significantly higher in GBS (median 220.8 pg/ml) than CIDP (17.3 pg/ml), multiple sclerosis (21.5 pg/ml), dementia (29.9 pg/ml) and healthy controls (5.6 pg/ml), Peak NfL levels also distinguished between controls and all other disease groups, but were not able to distinguish between CIDP, multiple sclerosis and dementia.

**Figure 4:**
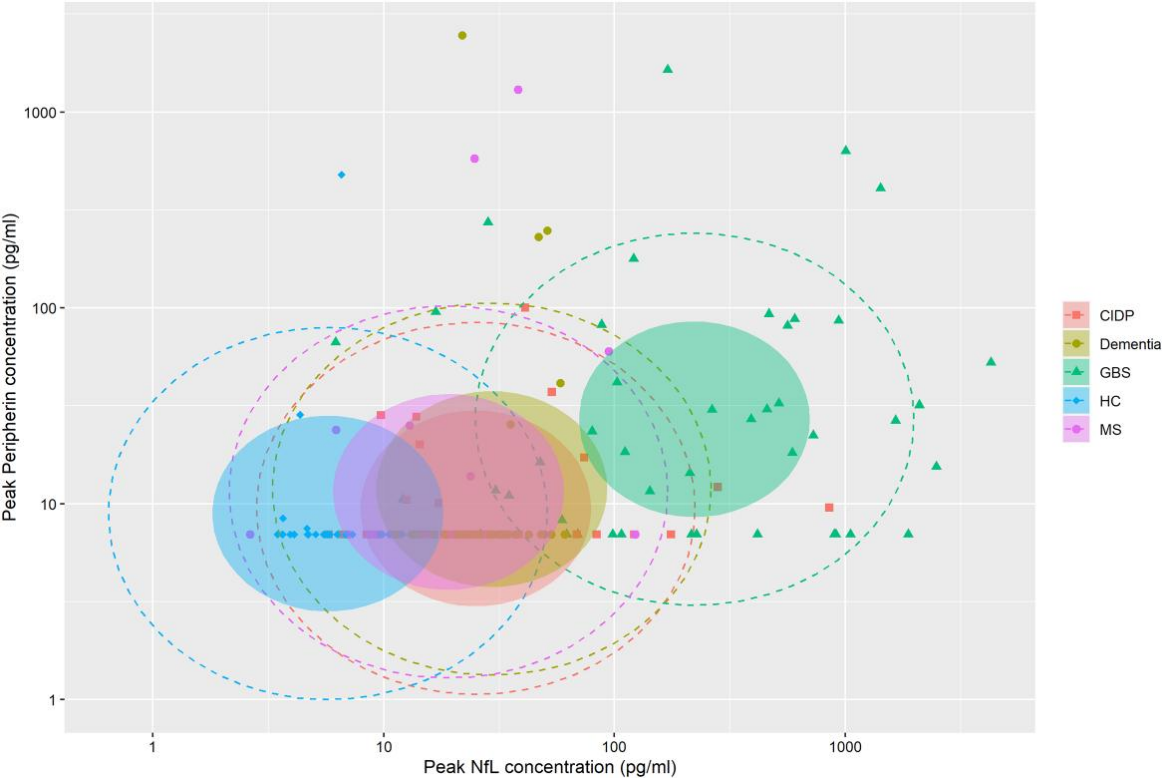
**Peak serum peripherin versus NfL concentration in different disease groups.** Coloured ellipses represent 50% confidence level for a multivariate *t*-distribution and dotted lines represent 95% confidence interval. CIDP = chronic inflammatory demyelinating polyradiculoneuropathy; GBS = Guillain-Barré syndrome; HC = healthy controls; MS = multiple sclerosis; NfL = neurofilament light chain.

Peak peripherin concentration showed a small positive association with peak NfL levels within the entire cohort (Spearman *rho* +0.40, *P* < 0.0001), but peripherin and NfL concentrations for each time point correlated poorly (Spearman *rho* +0.16, *P* = 0.0045), suggesting that peripherin and NfL display different dynamics within the serum. Figure 4 summarizes the relationship between peak peripherin and NfL within the different disease groups.

No associations were noted between peak peripherin levels and age, but higher peak NfL levels were associated with older age (Spearman *rho* +0.39, *P* < 0.0001) as is well described in the literature,^19^ suggesting that serum peripherin levels may vary less with age than NfL. Neither peripherin (*P* = 0.97) nor NfL (*P* = 0.45) displayed significant differences with patient gender.

#### Guillain-Barré syndrome

Twenty-five GBS patients had detailed clinical information available from serial measurements, and 85% of 21 Brighton classifiable patients had a diagnostic certainty of Brighton Level 1 or 2. In these 25 GBS patients the median time from symptom onset to nadir was 11 days. Five (20%) required intubation. The median Hughes GBS score on first assessment was 4 (bedridden/chair bound) and there was a median ONLS at nadir of 8. Of the nerve conduction studies performed (*n* = 21), 14 (67%) demonstrated conduction slowing, five (23%) were primarily axonal, and two (10%) mixed. There were no significant associations found between peak peripherin or NfL levels and MRC-SS, mRS, ONLS or iRODS at symptom nadir, nor were peak levels associated with presence of cranial neuropathy or autonomic disturbances, degree of areflexia or sensory disturbance, or neurophysiological subtype when Bonferroni correction was applied. Serial peripherin and NfL levels also showed no significant associations with MRC-SS, mRS, ONLS or iRODS at the same time point when adjusted for multiple comparisons. However, group sizes were small and therefore likely to be underpowered.

**Figure 5:**
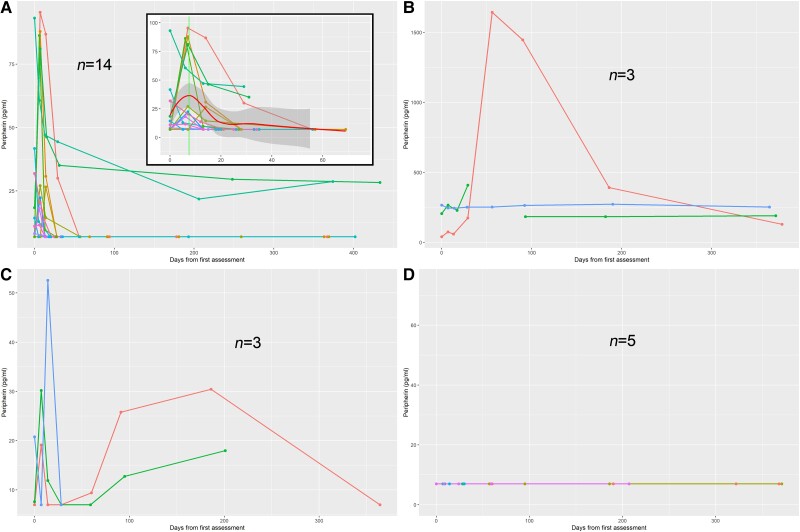
**Four patterns of longitudinal trends in serum peripherin with time (days from first assessment).** Serum peripherin trends following four distinct patterns: (**A**) single rise and fall, (**B**) persistent elevation with some fluctuation, (**C**) late second peak and (**D**) persistently normal. The *inset* for **A** displays a magnified picture of the initial 75 days from first assessment; the thick red line represents linear regression with loess smoothing and the shaded area represents the 95% standard error. The estimated peak of serum peripherin is at ∼Day 7 after first assessment.

Twenty-five of the 45 GBS patients had three or more time points of data available for analysis of individual trends. Serial peripherin levels conformed to four distinct longitudinal patterns (Fig. 5). The majority (14 patients, 56%) displayed a rise-and-fall pattern with the highest value either at first assessment (four patients) or within the first week of initial assessment (Fig. 5A). Local regression of this group (Fig. 5A, inset) using the loess smoothing estimated that peak peripherin levels occur at about 7 days after presentation/first assessment. In the small group of patients where a clear day of onset was recorded (*n* = 4), the highest serial peripherin level occurred between Day 6 and 18 of onset.

Smaller numbers of patients displayed other trends of serial peripherin concentration, with sustained elevated levels (Fig. 5B, three patients, 12%), variable high and low values (Fig. 5C, three patients, 12%), or levels below the LLOD throughout (Fig. 5D, five patients, 20%).

**Figure 6:**
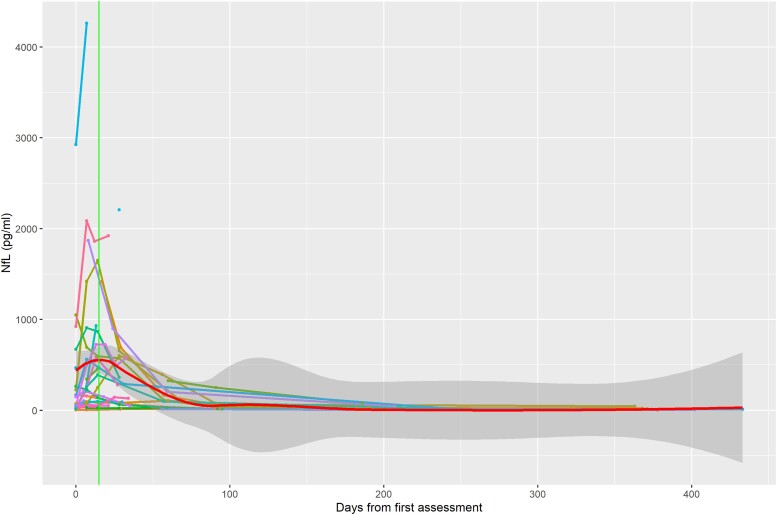
**Serum neurofilament light (NfL) longitudinal trends with time (days from first assessment).** Serum NfL trends broadly display a rise and fall pattern. The thick red line represents linear regression with loess smoothing and the shaded area represents the 95% standard error. The estimated peak of serum NfL is at ∼Day 16 after first assessment.

Figure 6 demonstrates the longitudinal trends of serial NfL concentrations in the same cohort of 25 GBS patients. This again displayed an expected rise-and-fall pattern, with local regression estimating that peak NfL levels tended to occur ∼16 days after first assessment.

#### Chronic inflammatory demyelinating polyradiculoneuropathy

The majority of the 35 patients in the CIDP cohort had typical CIDP (54%), with clinical outcome scores indicating stability or improvement on treatment on repeat assessment (Table 1). Peak peripherin and NfL concentrations displayed no significant association with disease phenotype, diagnostic certainty or treatment type. Peak NfL levels were higher in acute onset CIDP cases (mean NfL concentration 121.9 versus 16.3 pg/ml, *P* = 0.016). This was not noted for peak peripherin levels (*P* = 0.48).

Serial peripherin and NfL levels displayed no correlations with MRC-SS and i-RODS after Bonferroni adjustment for multiple comparisons.

#### Differentiating GBS from CIDP using serum peripherin and neurofilament light chain levels

**Figure 7:**
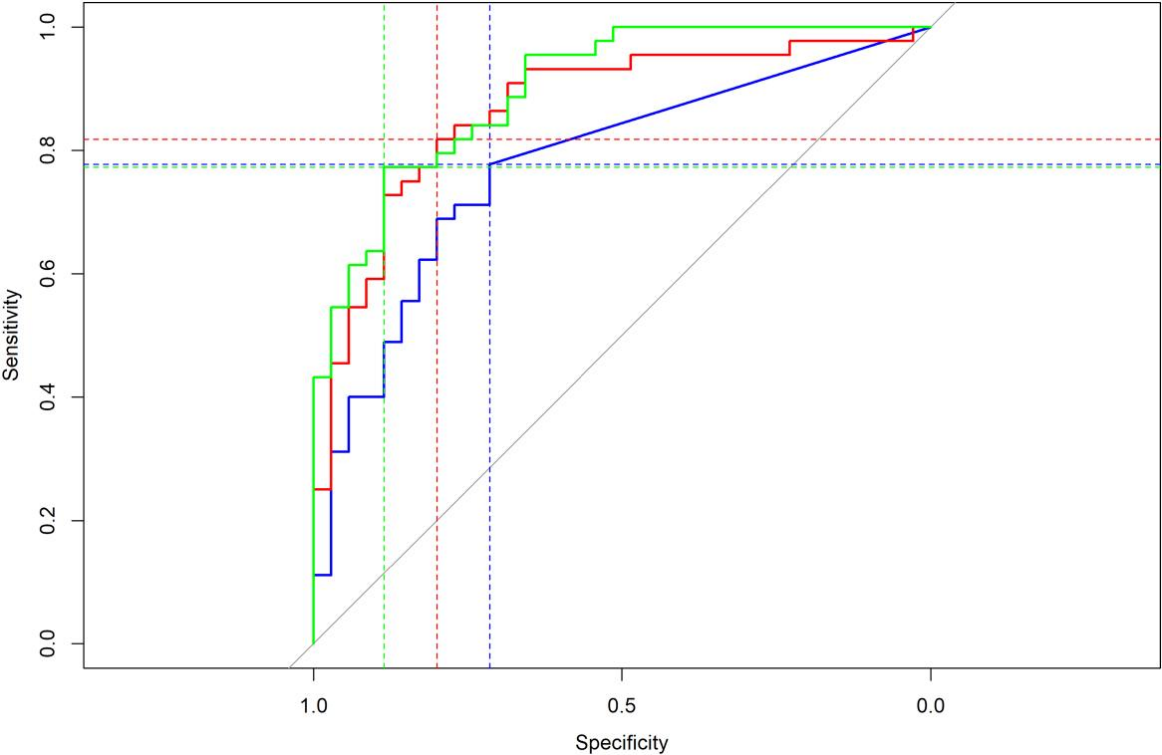
**Receiver operating characteristic curve using peak serum peripherin, neurofilament light (NfL) and a combined binomial regression model of peripherin and NfL to identify Guillain-Barré syndrome as opposed to CIDP**. Peak peripherin levels (using a threshold value of 6.98 pg/ml, the lower limit of detection for the assay) resulted in a sensitivity (Sn) of 0.78, specificity (Sp) of 0.71, area under the curve (AUC) of 0.78 (95% CI of 0.68–0.88) in identifying GBS over CIDP, resulting in a Youden index (Sp + Sn − 1) of 0.49. Peak NFL levels (threshold 56.3 pg/ml) yielded Sn = 0.82, Sp = 0.80, AUC = 0.87 (95% CI 0.79–0.95), and Youden index of 0.62. The combined model provided comparable Sn (0.77) and improved Sp (0.89), AUC (0.90, 95% CI = 0.84–0.97) and Youden index (0.66) compared to peak serum NfL or peripherin alone. CI = confidence interval; CIDP = chronic inflammatory demyelinating polyradiculoneuropathy; GBS = Guillain-Barré syndrome.

ROC curves were used to illustrate the diagnostic ability of peak serum peripherin and NfL in differentiating GBS from CIDP (Fig. 7). Using a cut-off of 6.98 pg/ml (the LLOD of the assay), peripherin displayed 78% sensitivity and 71% specificity in identifying GBS over CIDP (area under the curve, AUC = 0.78), whereas NfL with a cut-off value of 56.3 pg/ml displayed 82% sensitivity and 80% specificity (AUC = 0.87). However, analysis of a combined binomial regression model indicated that a combination of both peripherin and NfL peak values was superior to either value in isolation, with greatly improved specificity (89%) with retained sensitivity (77%) and AUC (0.90). Peripherin is superior at identifying processes in the periphery compared to NfL.

## Discussion

We have generated a highly sensitive Simoa-based immunoassay which measures serum peripherin as a biomarker of peripheral nerve axonal injury. Peripherin levels were raised significantly in patients with GBS compared to CIDP, CNS disease and healthy controls, where levels were most often low or undetectable. This peripherin assay is reproducible, reliable and has internal validity for its purpose. It demonstrates significant clinical advantages over the widely used NfL in being highly specific for peripheral nerve axonal injury as well as having dynamic sensitivity. It also does not seem to rise with older age, unlike NfL, simplifying interpretation of normative values without the need for age-specific cut-offs. Such findings require validation with standardized time points and larger groups.

Peripherin is abundant in peripheral nerves and should be a marker of peripheral axonal injury only. It is also found in lower amounts in the spinal cord, because of its presence in motor neuron cell bodies in the anterior horn and primary sensory afferents, as demonstrated by immunostaining. Our immunohistochemical findings are similar to existing online atlases detailing peripherin staining in mouse spinal cords.^20^ Peripherin in our spinal cord tissue homogenates would also be derived from proximal nerve roots collected during tissue harvesting. The amounts seen are very small. This may explain why some patients with multiple sclerosis have higher levels of peripherin, as these patients have spinal cord lesions and are likely to have damaged anterior horn cells or dorsal column axons. In contrast to peripherin, NfL is distributed throughout the PNS and CNS. Comparable detectable increases in serum or plasma NFL cannot distinguish PNS from CNS damage.^1,2^

The kinetics of the rise in peripherin *in vivo* are of interest and utility. Peripherin levels are significantly raised in the majority of GBS cases, peaking early after presentation and in most cases falling again as the disease improves (Fig. 5). Serum peripherin levels in GBS appear to peak quickly within 1–2 weeks from GBS onset in most cases, and fall to undetectable levels by ∼30 days, likely representing disease activity. The fall in most patients loosely appears to correlate with their recovery, and better than NfL. The pharmacokinetics of the rise and fall of peripherin followed the disease course of worsening and recovery in some cases more faithfully than NfL. We currently do not know the full kinetics of serum peripherin distribution and elimination, but it would appear to be shorter than NfL. At present we do not have enough data to indicate if the area under the peripherin curve or the peak rise correlates with outcome, and these data will be available from larger longitudinal cohorts with long-term outcome data.

Recent ideas of GBS being a spectrum of nodo-paranodopathy with varying degrees of paranodal and axonal damage determining the electrophysiological phenotype may be supported by these data.^21^ Although almost all GBS cases in the UK are ‘demyelinating’, the data here suggest peripherin consistently rises, indicating axonal damage in most cases. As the performance and sensitivity of the immunoassay is developed, the measurement of axonal damage prior to any detectable neurophysiological change may enable earlier identification of disease and guide more optimized treatment decisions.

Serum peripherin measurement should also be tested on cohorts of patients with other forms of axonal peripheral nerve disorders such as vasculitis, toxic and chemotherapy-induced neuropathies. This work is already underway.

Our data from the CIDP cohort, with low or undetectable peripherin levels, support the pathogenesis of CIDP being very different from GBS, with presumed primary myelin damage. The rate of axonal damage in CIDP, if it occurs, may be too low to be picked up by a biomarker with more dynamic characteristics. CIDP is a heterogenous condition and to fully understand the utility of peripherin, a large cohort needs to be studied, including a subset of severe and acute CIDP.

In some GBS cases, serum peripherin demonstrates a second rise. This may be due to a late secondary axonopathy, or perhaps secondary to early axonal regeneration. Expression of peripherin is upregulated in different neuronal types following injury, including the dorsal root ganglion and motor neurons following sciatic nerve trauma, supporting its role in neuronal regeneration.^8^ In other cases as well as in controls, there is a persistently high level of peripherin that does not change over time. The cause of this consistently raised peripherin level in this small number of patients is unexplained and will receive further attention in future work.

Serum NfL is far more ubiquitous than peripherin, and we would not expect NfL and peripherin to correlate exactly. We have shown that in peripheral nerve disease, raised levels of neurofilament and peripherin occur in parallel, but that there is an imperfect temporal correlation. Twelve patients with GBS had a raised NfL without any rise in peripherin, perhaps suggesting that there was significant damage to non-peripherin containing tissue. On the other hand, only three patients had a high peripherin with no significant rise in NfL in GBS, supporting peripherin as a sensitive marker of peripheral damage. Although peak NfL levels appear to be better at differentiating GBS from CIDP in our cohort than peripherin, a binomial regression of the two biomarkers improves model specificity, suggesting that peripherin does have a role alongside NfL in differentiating acute from chronic inflammatory neuropathies.

Peripherin has previously generated interest as a biomarker,^1,22–24^ but there have been few recent developments in its use, probably because the ultra-low serum levels made it difficult to measure. A recent study in MND indicated that raised peripherin was detectable in the blood of patients with MND using a commercial ELISA, but three orders of magnitude (>1000 times) higher than what we report.^9^ Future studies might consider a method comparison approach using Bland-Altman plots. The NfL data in our study, being in a similar range to what we report for peripherin, suggest that our data can be reasonably explained biologically.

The high peripherin levels in a small number of patients with multiple sclerosis, dementia and healthy controls were unexpected. The disease control samples were from highly phenotyped CNS disease cohorts, who could have had subclinical or non-reported peripheral neuropathy. Low-level peripherin expression has also been demonstrated in the cerebellum and corticospinal tract of the brainstem,^5,6^ and raised serum levels in such cases may represent CNS damage or atrophy. Peripherin is upregulated in rat models of traumatic brain injury,^25^ which suggests that larger degrees of CNS damage may also cause increases in peripherin. The normal controls are not known to have any peripheral neuropathy and these values need exploring in larger normal cohorts. For example, one individual initially collected as a healthy control had a markedly raised serum peripherin with normal NfL. On further questioning, this healthy control reported a history of foot drop after leg crossing, and transient paraesthesia, but had never sought medical advice. We were unable to contact the other patients with unexpectedly high levels, but this example is potentially illustrative. Biotin interference from multivitamin use can also lead to false positive results, which requires further exploration. Peripherin has a number of functions beyond simply axonal structure, including neurite outgrowth and stability, axonal transport and axonal myelination. It also interacts with proteins involved in vesicular trafficking, signal transduction, DNA/RNA processing, protein folding and mitochondrial metabolism,^4^ suggesting roles in alternative processes, which may cause unexplained rises beyond neuropathy alone. Further work will attempt to explain these rises.

In conclusion, we have demonstrated that peripherin is a new, dynamic and specific biomarker of peripheral nerve axonal damage, can differentiate acute nodo-paranodal axonal injury from slowly progressive demyelinating injury, and helps to distinguish central versus peripherally derived elevations in NfL by selectively rising in peripheral nerve injury. Further work will identify its relationship to long-term outcomes in GBS, and explain the identified controls and CNS diseases who have raised levels of peripherin without obvious explanation. Peripherin appears to be a novel and important biomarker in the search for biochemical markers of disease activity in neurology.

## Supplementary Material

awad234_Supplementary_Data

## Data Availability

Data are available on reasonable request to the corresponding author.
